# Melatonin inhibits TPA-induced oral cancer cell migration by suppressing matrix metalloproteinase-9 activation through the histone acetylation

**DOI:** 10.18632/oncotarget.8009

**Published:** 2016-03-09

**Authors:** Chia-Ming Yeh, Chiao-Wen Lin, Jia-Sin Yang, Wei-En Yang, Shih-Chi Su, Shun-Fa Yang

**Affiliations:** ^1^ Institute of Medicine, Chung Shan Medical University, Taichung, Taiwan; ^2^ Institute of Oral Sciences, Chung Shan Medical University, Taichung, Taiwan; ^3^ Department of Dentistry, Chung Shan Medical University Hospital, Taichung, Taiwan; ^4^ Department of Medical Research, Chung Shan Medical University Hospital, Taichung, Taiwan; ^5^ Whole-Genome Research Core Laboratory of Human Diseases, Chang Gung Memorial Hospital, Keelung, Taiwan

**Keywords:** melatonin, oral cancer, MMP, CREBBP, EP300

## Abstract

Melatonin exerts antimetastatic effects on liver and breast cancer and also inhibits matrix metalloproteinase (MMP) activity. However, the detailed impacts and underlying mechanisms of melatonin on oral cancer cell metastasis are still unclear. This study showed that melatonin attenuated the 12-O-tetradecanoylphorbol-13-acetate-induced migration of oral cancer cell lines, HSC-3 and OECM-1. Zymography, quantitative real-time PCR, and Western blotting analyses revealed that melatonin lessened MMP-9 enzyme activity as well as the expression of MMP-9 mRNA and protein. Furthermore, melatonin suppressed the phosphorylation of the ERK1/2 signalling pathway, which dampened MMP-9 gene transcription by affecting the expression of transcriptional coactivators, such as CREB-binding protein (CREBBP) and E1A binding protein p300 (EP300), and decreasing histone acetylation in HSC-3 and OECM-1 cells. Examinations on clinical samples exhibited that MMP-9, CREBBP, and EP300 were significantly increased in oral cancer tissues. Moreover, the relative level of CREBBP was positively correlated with the expression of MMP-9 and EP300. In conclusion, we demonstrated that melatonin inhibits the motility of HSC-3 and OECM-1 cells *in vitro* through a molecular mechanism that involves attenuation of MMP-9 expression and activity mediated by decreased histone acetylation.

## INTRODUCTION

Head and neck cancer is a common human cancer, and approximately 50% of cases occur in the oral cavity; more than 90% of these cases involve oral squamous cell carcinoma (OSCC) [1]. Cancer cell migration is increased by matrix degrading proteinases, integrins, and other cell adhesion molecules. Extracellular matrix (ECM) degradation is crucial in cancer invasion and metastasis because the metastasis of cancer cells requires the destruction of mesenchymal collagen or the spreading of the endothelial basement membrane to the surrounding tissue [2]. Thus, inhibition of ECM degradation by proteinases, such as matrix metalloproteinases (MMPs), and plasminogen activators is considered as critical in cancer therapy [3–5].

Numerous studies have revealed that MMPs are overexpressed in several types of malignant tumours, including oral cancer [6–8]. Of the MMPs, MMP-2 and MMP-9, known as the gelatinases that degrade the main constituent of the basement membrane and type IV collagen, have been recognised as crucial in cancer invasion and metastasis. Previous studies have revealed that inhibiting the activity of MMP-2 and MMP-9 reduces cancer cell metastasis [3, 5, 9–13]. However, the detailed effects of melatonin on oral cancer cell metastasis and MMP expression as well as the mechanisms by which these effects occur are still unclear.

Melatonin, also known as N-acetyl-5-methoxytryptamine, is synthesized from tryptophan via 5-hydroxytryptamine [14]. This hormone is crucial in regulating the immune system [15] and is a potent antioxidant agent [16, 17]. Other studies show that melatonin is a natural oncostatic agent, which protects humans from malignant neoplasms [18–20]. Previous studies have revealed that melatonin inhibits the growth and metastasis of breast cancer, cervical cancer, and ovarian cancer cells [21–23]. Recently, several studies have indicated that melatonin can decrease the expression of MMPs [24] and inhibit the invasion and metastasis of cancer cells [25–27]. For oral cancer, Goncalves et al. showed that melatonin inhibits cell viability in the oral carcinoma and inhibits the angiogenesis [28]. Reiter et al. also concluded that endogenously-produced and exogenously-applied melatonin are beneficial to the oral cavity [20]. The results indicate that melatonin in the oral cavity are likely to relate primarily to its anti-inflammatory and antioxidant activities [20]. However, few data regarding the antimetastatic potential of melatonin on oral cancer cells are available. Thus, this study examined the effects of melatonin with potential antimetastatic properties in 12-O-tetradecanoylphorbol-13-acetate (TPA)-treated HSC-3 and OECM-1 human oral cancer cells *in vitro* to investigate the signalling pathway of this process.

## RESULTS

### Effects of melatonin on the viability of HSC-3 and OECM-1 cells

**Figure 1:**
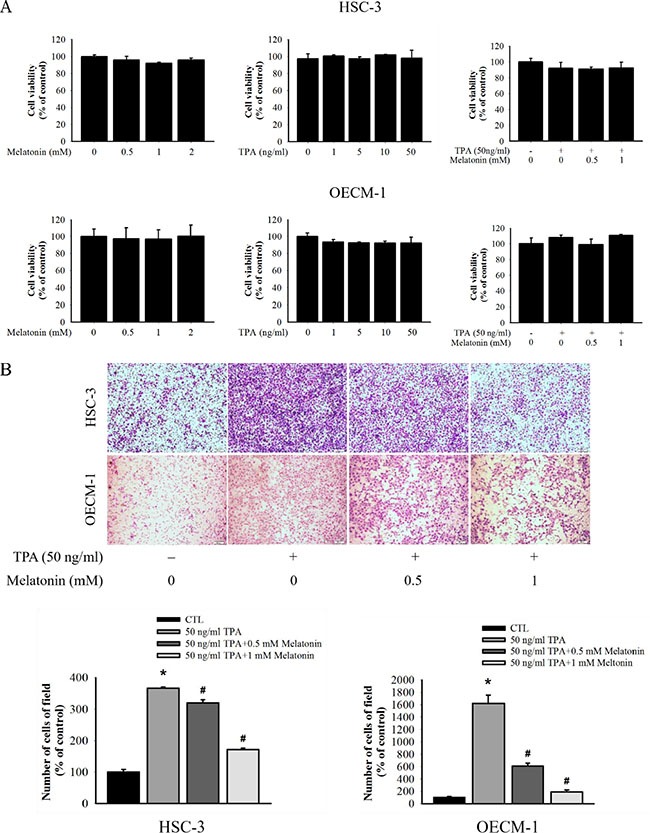
Effect of melatonin on cell migration in HSC-3 and OECM-1 cell (**A**) HSC-3 and OECM-1 cells were treated with TPA (0, 1, 5, 10, and 50 ng/mL) and melatonin (0, 0.5, 1, and 2 mM) for 24 h before a MTT assay for cell viability. The values are means ± SD of at least three independent experiments. (**B**) Cell migration was measured using transwell for 24 h (OECM-1 cell) and 48 h (HSC-3 cell) with polycarbonate filters, respectively. The migration abilities of HSC-3 and OECM-1 cells were quantified by counting the number of cells that invaded the underside of the porous polycarbonate, as described in the Materials and Methods. The values represent the means ± SD of at least three independent experiments. *p < 0.05 compared with the vehicle group; ^#^*p* < 0.05 compared to the TPA treatment group.

We measured cell viability by using various concentrations (0, 0.5, and 1 mM) of melatonin for 24 h by MTT assay to investigate the cytotoxicity of melatonin on HSC-3 and OECM-1 cells. Melatonin demonstrated no significant toxicity on the TPA-treated and untreated HSC-3 and OECM-1 cells at concentrations between 0 and 1 mM for 24 h (Figure 1A). The range of concentrations was explored in subsequent experiments.

### Effects of melatonin on migration of HSC-3 and OECM-1 cells

The antimetastatic activity of melatonin on HSC-3 and OECM-1 was measured through the migration assay by using the transwell. The results show that TPA treatment resulted in a noticeable increase in cell migration, whereas melatonin inhibited the TPA-induced cell migration in a dosedependent manner (Figure 1B). Collectively, these results indicate that melatonin effectively prevented TPA-induced migration in the HSC-3 and OECM-1 cells.

### Effects of melatonin on MMP-9 enzyme activity, protein expression, and mRNA expression

**Figure 2:**
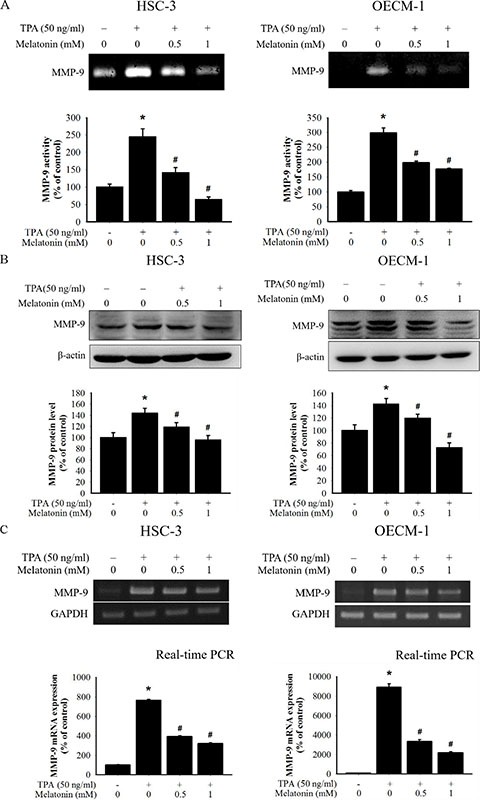
Effects of TPA and melatonin on MMP-9 activity, protein, and mRNA level (**A**) HSC-3 and OECM-1 cells were treated with TPA (50 ng/mL) and melatonin (0, 0.5, and 1 mM) for 24 h and then subjected to gelatin zymography analysis to determine the activities of MMP-9, (**B**) or western blotting to analyze the protein levels of MMP-9. (**C**) The mRNA expression of MMP-9 was analyzed by RT-PCR and Quantitative real time-PCR (qPCR). The values represented the means ± SD of at least three independent experiments. *p < 0.05 compared to the vehicle group; ^#^*p* < 0.05 compared to the TPA treatment group.

The gelatin zymography assay was used to investigate the effect of melatonin against the MMP-9 enzymatic activity in HSC-3 and OECM-1 cells following TPA treatment. Melatonin was found to significantly reduce TPA-induced MMP-9 gelatinolytic activity through gelatin zymography (Figure 2A). The results also demonstrated that melatonin treatment resulted in a reduction in TPA-induced intracellular expression of MMP9 (Figure 2B). Reverse transcription polymerase chain reaction (RT-PCR) and quantitative real time-PCR (qPCR) was then used to investigate the effect of melatonin treatment on the regulation of TPA-induced MMP9 transcription. Melatonin treatment resulted in a reduction in the MMP9 mRNA expression levels in a dosedependent manner (Figure 2C). QPCR also demonstrated a TPA-induced increase in MMP-9 mRNA expression in HSC-3 and OECM-1 cells as well as suppression of this increase for melatonin treatment. These results indicate that melatonin suppresses TPA-induced MMP-9 expression at the protein and mRNA levels and that the compound inhibits the enzymatic activity of MMP-9.

### Effects of melatonin on MAPK pathways

**Figure 3:**
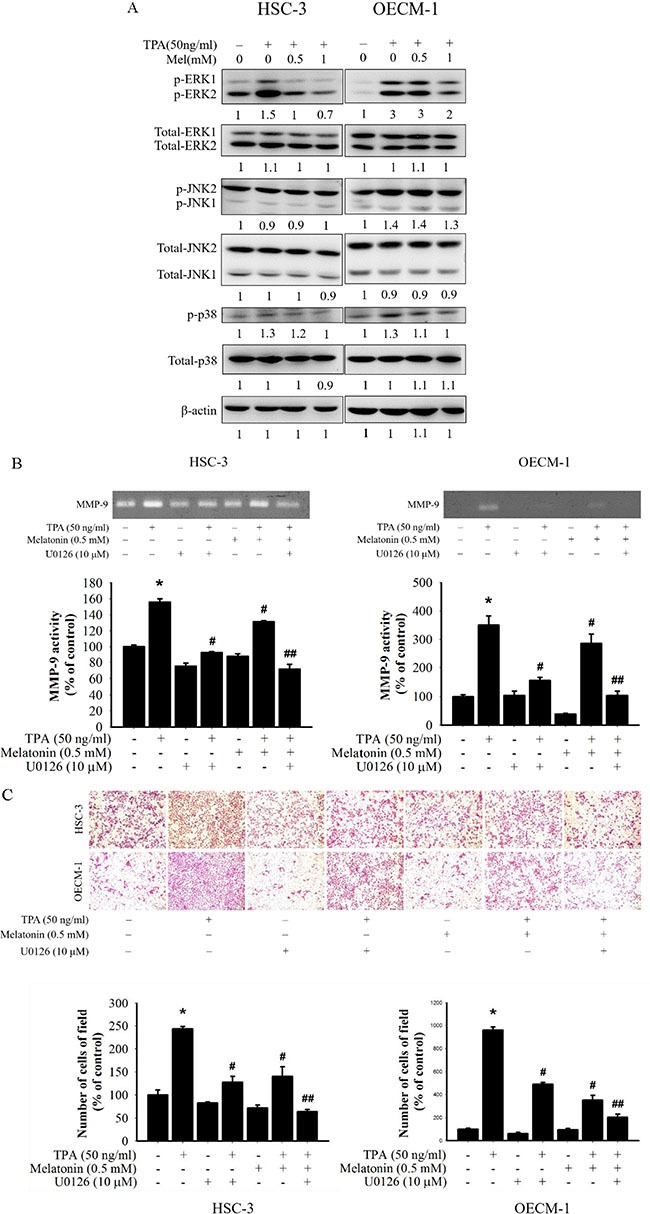
Effects of TPA and melatonin on the MAPK pathway (**A**) HSC-3 and OECM-1 cells were treated with TPA (50 ng/mL) and melatonin (0, 0.5, 1 mM) and then subjected to Western blotting to analyze the levels of ERK 1/2, JNK, and p38. (**B**) HSC-3 and OECM-1 cells were pre-treated with U0126 (10 μM) for 1 h and then incubated in the presence or absence of TPA (50 ng/mL) and melatonin (0.5 mM) for 24 h. The medium was subjected to gelatin zymography. (**C**) Cell migration was measured using transwell for 24 h (OECM-1 cell) and 48 h (HSC-3 cell) with polycarbonate filters, respectively. The migration abilities of HSC-3 and OECM-1 cells were quantified by counting the number of cells that invaded the underside of the porous polycarbonate. The values represented the means ± SD of at least three independent experiments. *p < 0.05 compared to the vehicle group; ^#^*p* < 0.05 compared to the TPA treatment group; ^##^*p* < 0.05 compared to the combination of TPA and U0126, or melatonin treatment group.

After the inhibitory effects of melatonin on cell migration and MMP-9 expression were revealed, the effects of melatonin on the expression of mitogen activated protein kinase (MAPK) pathways were investigated to elucidate their underlying mechanisms. Western blotting revealed that TPA significantly increased the phosphorylation of three MAPK pathways in HSC-3 and OECM-1 cells. Furthermore, melatonin reduced the phosphorylation of ERK1/2 in HSC-3 and OECM-1 cells, but not the phosphorylation of the JNK and p38 pathways (Figure 3A). To further determine whether melatonin inhibition of MMP-9 activity was caused mainly by the inhibition of the ERK1/2 signalling pathway, the effects of melatonin on a specific inhibitor of the ERK1/2 (U0126) in HSC-3 and OECM-1 cells were investigated. In the gelatin zymography assay, TPA-induced MMP-9 activity of HSC-3 and OECM-1 cells was significantly reduced by the ERK1/2 inhibitor (U0126) (Figure 3B), and the result of the migration assay was similar to that of the gelatin zymography assay (Figure 3C). Moreover, these results revealed that a combined treatment of the ERK1/2 inhibitor (U0126) and melatonin further reduced MMP-9 expression. Thus, inhibiting ERK1/2 signalling pathways might result in reduced MMP-9 expression.

### Melatonin does not inhibit the activation of the NF-κB pathways

**Figure 4:**
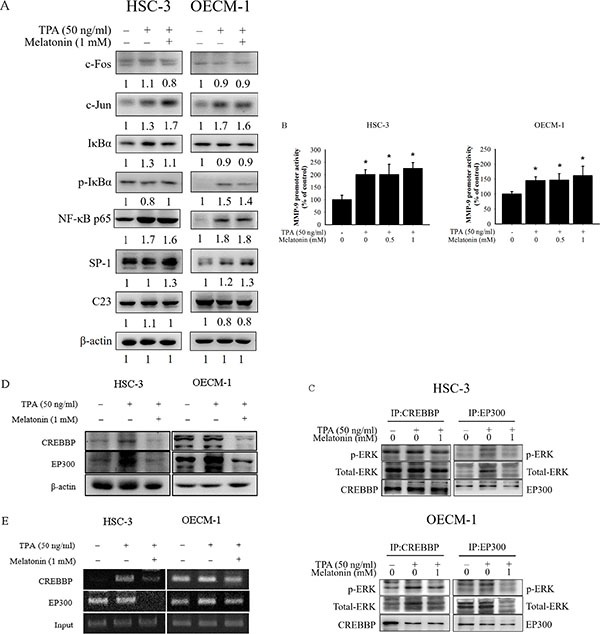
Effects of TPA and melatonin on the transcriptional factors and MMP-9 promoter activity (**A**) HSC-3 and OECM-1cells were treated with TPA (50 ng/mL) and melatonin (1 mM) for 24 h and then the nuclear fraction or total cell lysate was analyzed by western blotting. Levels of c-Fos, c-Jun, IκBα, NF-κB p65, and SP-1 from the nucleus or total cell lysate were immunodetected with c-Fos, c-Jun, IκBα, NF-κB p65, and SP-1-specific antibodies, respectively. (**B**) HSC-3 and OECM-1 cells were transfected with the transcriptional factors binding sites MMP-9-Luc. The transfected cells were treated with TPA (50 ng/mL) and melatonin (0, 0.5, and 1 mM) for 24 h. Luciferase activity, determined in triplicates, was normalized to β-galactosidase activity. The values represented the means ± SD of at least three independent experiments. *p < 0.05 compared to the vehicle group. (**C**) Nuclear extracts were immunoprecipitated (IP) with CREBBP and EP300 antibodies to analyze the interaction between ERK1/2 and CREBBP, or EP300 *in vivo*. Protein expressions were evaluated by western blotting. (**D**) The protein levels of CREBBP and EP300 were analyzed by western blotting. (**E**) ChIP analysis of the association of transcription coactivators, CREBBP and EP300, with the MMP-9 promoter region in HSC-3 and OECM-1 cells.

Previous studies have demonstrated that transcription factor NFκB, AP-1, and SP-1 are crucial in TPA-induced MMP9 gene transcription [29]. Thus, the effects of melatonin on the NFκB, AP-1, and SP-1 signalling pathways were assessed in this study. The Western blotting analysis demonstrated TPA-induced phosphorylation of IκBα in OECM-1 cells, but this was not prevented by melatonin treatment (Figure 4A). In addition, melatonin did not reduce the phosphorylated NFκB p65, AP-1 and SP-1 induced by TPA (Figure 4A). Moreover, the effect of melatonin on the binding of these transcription factors to MMP-9 promoters, which was measured using the luciferase report assay, was not obvious (Figure 4B). Some studies have indicated that ERK1/2 signalling increases the expression of transcriptional coactivator CREB-binding protein (CREBBP) and its paralog E1A-binding protein (EP300) [30, 31]. To further determine the coactivator factor, CREBBP and EP300, which participate in regulating MMP-9 transcription through ERK1/2 signalling, the immunoprecipitation assay was used. The results revealed that activated ERK1/2 had binding affinities to EP300 and CREBBP protein and melatonin significantly repressed the binding affinities (Figure 4C). Moreover, the results of Western blotting showed that the expression of TPA-induced CREBBP and EP300 were inhibited by melatonin (Figure 4D). We further performed the chromatin immunoprecipitation (ChIP) assay to investigate the involvement of these coactivators in the transcriptional inhibitory effects of melatonin on MMP-9. As illustrated in Figure 4E, the binding ability of CREBBP and EP300 to the MMP-9 promoter was decreased in HSC-3 and OECM-1 cells following melatonin treatment.

**Figure 5:**
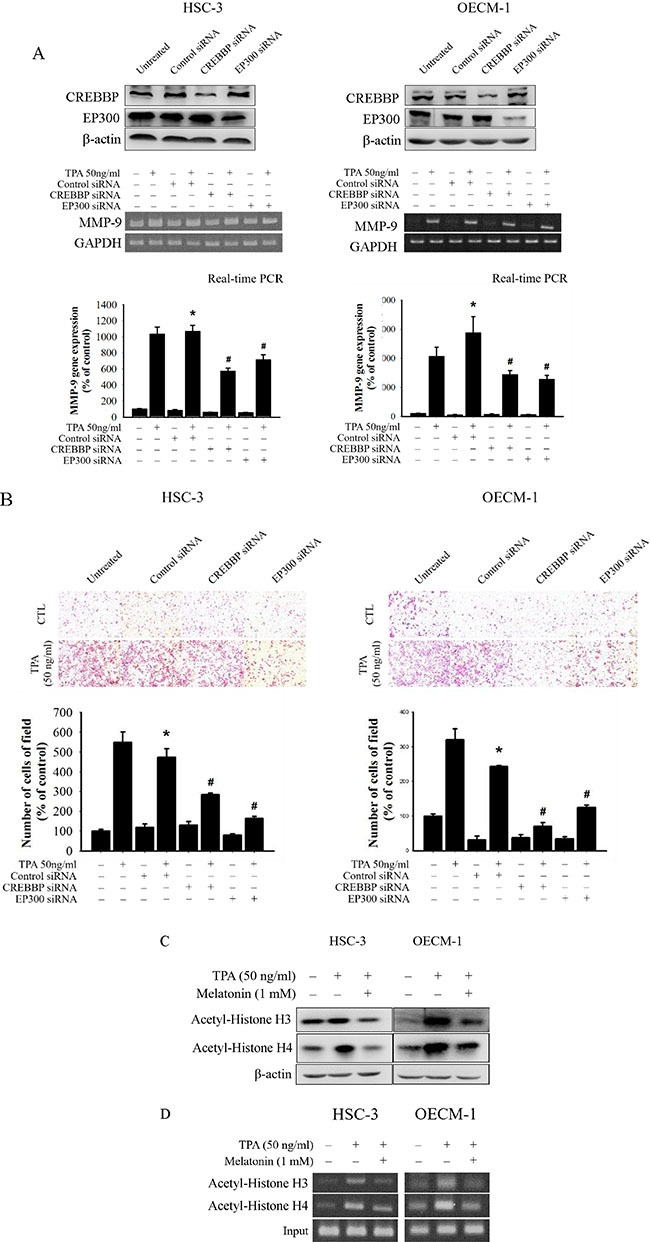
Critical role of CREBBP and EP300 in TPA-induced transcriptional inhibition of MMP-9 in HSC-3 and OECM-1 cells (**A**) HSC-3 and OECM-1 cells were transfected with the CREBBP and EP300 siRNA, and then treated with TPA (50 ng/mL) for 24 h. MMP-9 mRNA levels were determined by RT-PCR and real-time PCR. (**B**) Cell migration was measured using transwell for 24 h (OECM-1 cell) and 48 h (HSC-3 cell) with polycarbonate filters, respectively. The values represented the means ± SD of at least three independent experiments. *p < 0.05 compared to the vehicle group; ^#^*p* < 0.05 compared to the TPA treatment group. (**C**) HSC-3 and OECM-1 cells were treated with TPA (50 ng/mL) and melatonin (1 mM) and then subjected to Western blotting to analyze the levels of Ac-H3 and Ac-H4. (**D**) ChIP analysis of the association of histone H3 and H4 acetylation with the MMP-9 promoter region in HSC-3 and OECM-1 cells.

To further determine whether melatonin inhibition of MMP-9 expression and oral cancer cell migration was caused mainly by the inhibition of the CREBBP/EP300 signalling pathway, the effects on a specific siRNA of CREBBP and EP300 in HSC-3 and OECM-1 cells were investigated. In the RTPCR assay, TPA-induced MMP-9 mRNA expression of HSC-3 and OECM-1 cells was significantly reduced by the CREBBP and EP300 siRNA (Figure 5A), and the result of the migration assay was similar to that of the RT-PCR assay (Figure 5B). These results indicate that melatonin suppresses TPA-induced MMP-9 expression by reducing the CREBBP/EP300 but not NF-κB signalling pathway in HSC-3 and OECM-1 cells.

Previous studies have demonstrated that CREBBP/EP300 acetylates histones, resulting in chromatin remodelling and relaxation of the chromatin structure to enable transcription [32, 33]. Thus, the effects of melatonin on histone acetylation were also assessed in this study. Western blotting revealed that TPA significantly increased the histone H3 and H4 acetylation while melatonin reduced the acetylation of histone H3 and H4 in HSC-3 and OECM-1 cells (Figure 5C). Furthermore, the binding of the acetylation of histone H3 and H4 to the MMP-9 promoter decreased in HSC-3 and OECM-1 cells following melatonin treatment by ChIP assay (Figure 5D). Overall, these findings indicated that melatonin might induce transcriptional inhibition of MMP-9 in HSC-3 cells by suppressing the CREBBP and EP300 expression and histone acetylation.

### CREBBP and EP300 are the key regulators for the transcriptional inhibition of MMP-9 through histone acetylation by melatonin

**Figure 6:**
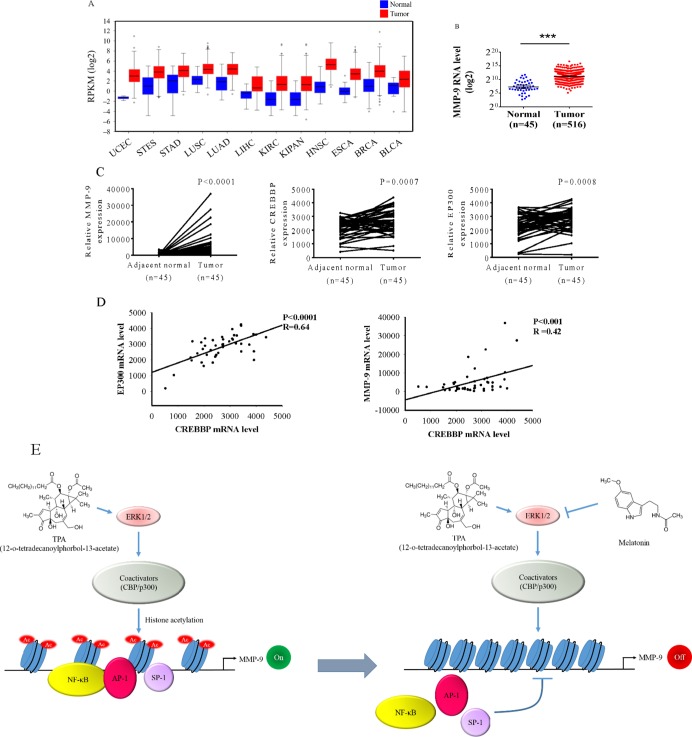
Levels of MMP-9, CREBBP, and EP300 are increased in head and neck squamous cell carcinoma samples (**A**) The expression of MMP-9 among 12 cancer types from The Cancer Genome Atlas (TCGA) Data Portal from Broad GDAC Firehose data portal. The mRNA RPKM (Reads per Kilobase of exon model per Million) reads per million mappable reads of all samples were selected and analyzed for comparing abundances by the website of Broad GDAC Firehose. (**B**) The expression of MMP-9 in normal and head and neck squamous cell carcinoma from TCGA Data Portal. ***p < 0.05. (**C**) Relative expression of MMP-9, CREBBP, and EP300 in 45 pairs of head and neck squamous cell carcinoma tumor tissues and their corresponding adjacent non-cancerous tissues. (**D**) The correlations among mRNA levels of CREBBP and EP300 as well as MMP-9 in head and neck squamous cell carcinoma. A significant correlation was found between CREBBP and EP300 (Spearman rank correlation coefficient *r* = 0.64, *p* < 0.001) as well as MMP-9 (*r* = 0.46, *p* < 0.001). (**E**) A model depicting reciprocal regulation between melatonin and MMP-9 transcription in oral cancer. UCEC: uterine corpus endometrial carcinoma. STES: stomach and esophageal carcinoma. STAD: stomach adenocarcinoma. LUSC: lung squamous cell carcinoma. LUAD: lung adenocarcinoma. LIHC: liver hepatocellular carcinoma. KIRC: kidney renal clear cell carcinoma. KIPAN: pan-kidney cohort. HNSC: head and neck squamous cell carcinoma. ESCA: esophageal carcinoma. BRCA: breast invasive carcinoma. BLCA: bladder cancer.

To further support our findings, we examined the MMP-9 expression in The Cancer Genome Atlas (TCGA) Data Portal from Broad GDAC Firehose to determine whether MMP-9 was involved in the development of head and neck squamous cell carcinoma (HNSCC) and found that MMP-9 exhibits a higher expression in tumours than in various tumours (Figure 6A). The MMP-9 expression was significantly increased in cancer tissue compared with that in the normal parts in HNSCC (Figure 6B). To further support this conclusion, we also examined the expression of MMP-9, CREBBP, and EP300 in HNSCC tissue and their corresponding noncancerous tissue by using the TCGA Data Portal. The results demonstrated that MMP-9, CREBBP, and EP300 were significantly increased in HNSCC tissue (Figure 6C). Moreover, the relative CREBBP levels were positively correlated with the expression of MMP-9 and EP300 mRNAs in HNSCC. A significant correlation was found between CREBBP and EP300 (Spearman rank correlation coefficient *r* = 0.64, *p* < 0.001) as well as MMP-9 (*r* = 0.46, *p* < 0.001) (Figure 6D).

## DISCUSSION

Numerous studies have revealed that melatonin possesses antitumour effects, including regulating cancer cell apoptosis, angiogenesis, and metastasis [34, 35]. The antitumour and antimetastatic activities of melatonin against human cancer cells have been demonstrated previously; however, few data regarding the effects of melatonin on oral cancer metastasis and the mechanisms of these effects are available. We demonstrated that melatonin inhibits migration of HSC-3 and OECM-1 oral cancer cells, inhibits the gene expression and enzyme activity of MMP-9, inhibits phosphorylation of ERK1/2, inhibits the expression of CREBBP and EP300 transcription factors, and reduces histone acetylation on the MMP-9 gene.

Metastasis involves complex and redundant pathways, including the microenvironmental interaction at the primary site, ECM degradation and growth at a distant site [36]. In this study, melatonin inhibited the migration of HSC-3 and OECM-1 oral cancer cells (Figure 1B). The critical step for invasion and metastasis is the breakdown of the basement membrane, which requires the activation of proteolytic enzymes, such as MMPs [37, 38]; Previous studies have revealed that downregulation, the activity of MMPs, can provide early targets for preventing cancer metastasis [9, 39]. In this study, the MMP-9 expression was induced by TPA, a potent tumour promoter used for studying cancer metastasis that can induce MMP-9 expression and promote cancer cell metastasis [40]. The zymography and Western blotting data in this study indicated that melatonin significantly inhibited the MMP-9 enzyme activity and protein expression in HSC-3 and OECM-1 cells (Figure 2A and 2B). Our results indicated that MMP-9 is a critical target of melatonin for regulation of oral cancer metastasis.

MAPK is a family of serine/threonine kinases, including ERK1/2, JNKs, and p38. Activation of MAPKs is followed by phosphorylation of various cytosolic substrates that participate in numerous cellular activities, such as cell proliferation, differentiation, invasion, migration, and death [41]. Activating the MAPK pathway is crucial for inducting MMP-9 expression in various cancer cell lines [42, 43], and suppressing the MAPK signalling pathway can reduce MMP expression [5, 44]. This study revealed that melatonin inhibits the TPA-induced MMP-9 expression of oral cancer HSC-3 and OECM-1 cells through ERK1/2 phosphorylation (Figure 3A). However, Mao et al. revealed that melatonin inhibits breast cancer cell invasion by downregulating the p38 pathway [45]. Moreover, Zhou et al. found that melatonin inhibits the migration of human lung adenocarcinoma cell lines involving JNK1/2 pathway [46]. Therefore, we speculate that the effects of melatonin on MMP-9 can differ depending on the stimulus and the cancer cell line.

Regulation of MMP-9 expression occurs primarily through transcriptional, posttranscriptional, protein modification and cell surface localisation [29, 47]. Previous studies have indicated that MMP-9 inhibition is followed by the regulation of MMP-9 promoter activity by transcription factors, such as NF-κB, AP-1, and SP-1 [29, 48–50]. In this study, melatonin significantly inhibited MMP-9 mRNA expression (Figure 2C). However, the activity of the MMP-9 promoter and its transcriptional factors was not inhibited by melatonin (Figure 4A and 4B). The results of this study indicated that the TPA-induced MMP-9 melatonin inhibition may not have occurred through transcription factors (NF-κB, AP-1, and SP-1) but through other transcriptional mechanisms. Gene transcription is regulated by sequence-specific transcription factors and the interaction of transcription factors and coactivators [51]. Previous studies have observed that the regulation of histone acetylation by the CREBBP and EP300 transcription coactivators is associated with MMP-9 gene transcription [52, 53]. Moreover, Ma et al. reported that histone H3 and H4 acetylation is linked to the transcriptional activation of MMP-9, which is driven by mitogen signalling in HeLa cells [54]. Thus, CREBBP and EP300 may be crucial in MMP-9 gene transcription [52, 53]. Moreover, previous studies have observed that ERK1/2 pathways are associated with regulating the expression of transcriptional coactivators CREBBP and EP300 [30, 31]. Therefore, we hypothesise that melatonin inhibits TPA-induced MMP-9 expression in oral cancer cells by regulating the coactivator family through the ERK1/2 signalling pathway. The immunoprecipitated assay revealed that melatonin-repressed ERK phosphorylation connects to the CREBBP/EP300 coactivator family (Figure 4C), and the Western blotting and ChIP results demonstrated that the expression of the CREBBP/EP300 coactivator family was inhibited by melatonin (Figure 4D and 4E).

Melatonin is used widely for the concerning the daily cycle of light and darkness to body structures and possesses anti-aging effects [55–57]. It is achievable for 20 mM melatonin clinically [58]. Moreover, a systematic review article also concluded that time to maximal plasma/serum concentration (Tmax) was approximately 50 min following oral immediate-release formulations of melatonin and the bioavailability of oral melatonin was approximately 15% [59]. Thus, the experimental concentrations of Melatonin used in this study (0–1 mM) could become clinically achievable. One limitation of the present study was the lacking of animal study, which could provide additional support to our findings in this study. Moreover, the physiological relevance of the experimental concentrations of melatonin used in the *in vitro* study may not be calculated accurately *in vivo* and will be included in our future work.

In summary, our results reveal that transcriptional suppression of the MMP-9 gene occurred through the downregulation of ERK1/2 signalling pathways, resulting in the decreased expression of CREBBP and EP300, which inhibited histone acetylation on the MMP-9 promoter (Figure 6E). Furthermore, these mechanisms interfere with the assembly of functional transcription complexes on the MMP-9 promoter, but the recruitment of transcription factors, such as NF-κB, AP-1, and Sp-1, is not affected by melatonin. Thus, inhibition of oral cancer metastasis by melatonin can provide crucial preventive and therapeutic protection against oral cancer.

## MATERIALS AND METHODS

### Cell and cell culture

Human oral squamous cell carcinoma (OSCC) cell line HSC-3 cells were purchased from and validated by the Japanese Collection of Research Bioresources Cell Bank (JCRB, Shinjuku, Japan) and were cultured in DMEM/F-12 or RPMI-1640 medium (Life Technologies, Grand Island, NY), 10% fetal bovine serum (FBS), 2 mM glutamine, 100 U/ml penicillin, and 100 μg/ml streptomycin. Oral epidermal carcinoma cell line OECM-1 cells were obtained from Dr Meng's group where the cell line is originally established and authenticated [60] and maintained in RPMI (Gibco) supplemented with 10% FBS. All cell cultures were maintained at 37°C in a humidified atmosphere of 5% CO2. For melatonin treatment, appropriate amounts of stock solution of melatonin were added into the culture medium to achieve the indicated concentrations. The cells were then incubated for the indicated time periods. Dimethyl sulfoxide solution without melatonin was used as blank reagent.

### Analysis of cell viability (MTT assay)

To evaluate the cytotoxicity of melatonin, an MTT colorimetric assay was performed to determine cell viability. OECM-1 and HSC-3 were seeded in 24-well plates at a density of 9 × 10^4^ or 1.2 × 10^5^ cells per well and treated with 0, 0.5 and 1 mM of melatonin at 37°C in 5% CO2 for 24 h. At the end of the exposure period, the cells were washed with PBS and incubated with 0.8 mL of MTT (Sigma chemical Co., St. Louis, MO, USA) per well (final concentration, 0.5 mg/mL) at 37°C in 5% CO2 for 4 h. The viable cell number was directly proportional to the production of formazan following solubilization with isopropanol, which was measured spectrophotometrically at 563 nm (Beckman Spectrophotometer DU640, Beckman Instruments, and Fullerton, CA, USA).

### Cell migration assays

Cell migration was assayed according to the methods described by Lin et al. [3]. After treatment with for 24 h, the surviving HSC-3 and OECM-1 cells were harvested and seeded to a Transwell (Costar, Corning, NY, USA) at 4 × 10^4^ cells per well in 0.5% fetal bovine serum medium, and then incubated for 24 h or 40 h at 37°C. The migrating cells were fixed with methanol and stained with Giemsa. The cell numbers were counted by light microscopy.

### Determination of MMP-9 activity by zymography

The activities of MMP-9 in the conditional medium were measured by gelatin zymography protease assays as previously described [61]. Briefly, collected media of an appropriate volume were prepared with SDS sample buffer without boiling or reduction, and subjected to 0.1% gelatin–8% SDS-PAGE electrophoresis. After electrophoresis, the gels were washed with 2.5% Triton X-100 and incubated in a reaction buffer (40 mM Tris–HCl, pH 8.0; 10 mM CaCl2 and 0.01% NaN3) at 37°C for 12 h. The gel was stained with Coomassie brilliant blue R-250 for visualization.

### Western blotting analysis for determining molecular pathway

Total cell lysates or nuclear extracts were prepared as previously described [3]. The cell lysates were separated in a 10% polyacrylamide gel and transferred onto a nitrocellulose membrane. The blot was subsequently incubated with 5% non-fat milk in Tris-buffered saline (20 mM Tris, 137 mM NaCl, pH 7.6) for 1 h to block non-specific binding, and then overnight with polyclonal antibodies against three MAPKs (ERK 1/2, JNK 1/2, and p38), CREBBP, EP300, acetyl-Histone-H3, acetyl-Histone-H4 and β-actin with the specific antibodies for unphosphorylated or phosphorylated forms. The blots were then incubated with horseradish peroxidase goat anti-rabbit or anti-mouse IgG for 1 h. Signal was detected by using an enhanced chemiluminescence (ECL) commercial kit (Amersham Biosciences, Piscataway, NJ, USA). The relative photographic density was quantitated by scanning the photographic negatives on a gel documentation and analysis system (AlphaImager 2000, Alpha Innotech Corporation, and San Leandro, CA, USA).

### RNA preparation, semi-quantitative RT-PCR, TaqMan quantitative real-time PCR

Total RNA was isolated from cancer cells using Total RNA mini kit (Geneaid). For Semi-quantitative RT-PCR, the PCR was performed in a reaction mixture containing 2 μL cDNA, 0.2 mM dNTP mixture, 2 μM of each primers, 1 U Taq DNA polymerase, and 1-fold concentration of Thermal Pol Buffer (New England BioLabs, MA, USA). The specific primer sequences for these genes are as following: MMP-9: 5′-CAACATCACCTATTGGATCC-3′ (forward), 5′- CGG GTGTAGAGTCTCTCGCT-3′(reverse), and GAPDH: 5′- AGCCTTCTCCATGGTTGGTGAAGAC-3′ (forward), 5′- CGGAGTCAACGGATTTGGTCGTAT-3′ (reverse). Moreover, quantitative real-time PCR analysis was performed using TaqMan one-step PCR Master Mix (Applied Biosystems, Foster City, CA, USA). Total cDNA (2 μg) was added per 9 μl reactions with MMP-9 or GAPDH primers and TaqMan probes. The MMP-9 (Hs00234579_m1) and GAPDH (Hs99999905_m1) primers and probes were designed using commercial software (ABI PRISM Sequence Detection System; Applied Biosystems, Foster City, CA, USA). Quantitative real-time PCR assays were conducted in triplicate on a StepOnePlus sequence detection system. Threshold was set above the non-template control background and within the linear phase of target gene amplification to calculate the cycle number at which the transcript was detected [3].

### Transfection and MMP-9 promoter-driven luciferase assays

The HSC-3 and OECM-1 cells were seeded at a concentration of 10^5^ or 1.4 × 10^5^cells per well in 24-well cell culture plates. After 12 h of incubation, pGL3-basic (vector) and MMP-9 promoter plasmid were co-transfected with a β-galactosidase expression vector (pCH110) into cells using Turbofect (Fermentas, Carlsbad, CA) as previously described [5]. After 24 h of transfection, the cells were treated with vehicle or melatonin (0–1 mM) for 24 h. The cell lysates were harvested and luciferase activity was determined using a luciferase assay kit. The value of the luciferase activity was normalized to transfection efficiency and monitored by β-galactosidase expression.

### Chromatin immunoprecipitation analysis (ChIP)

Chromatin immunoprecipitation analysis was performed as described previously by Yang et al. [5]. In brief, chromatin and proteins from approximate 1 × 10^6^ cells were crosslinked with 1% formaldehyde for 10 min at room temperature. These cells were collected, lysed, and sonicated on ice to shear the chromatin DNA to a length between 200–1000 base pair by using Sonicator 3000 (Misonix, NY, USA). DNA immunoprecipitated with anti-CREBBP, anti-EP300, anti-acetyl-histone-H3 or anti-acetyl-histone-H4 was purified and extracted using PCR purification kit (QIAGEN, Redwood City, CA, USA).

### Immunoprecipitation assay (IP)

Immunoprecipitation analysis was modified as described previously by Meissner et al. [31]. In brief, cell lysates were immunoprecipitated with 50 μl of Protein A conjugated with CREBBBP and EP300 antibodies affinity matrix-conjugated individually in NET-N buffer [1 M Tris-Hcl (pH 8.0), 0.5 M EDTA, 5 M Nacl, and 0.5% Nonidet P-40] under gentle shaking at 4°C overnight. Immunoprecipitated beads were pelleted and washed three times with 1 ml of NET-N buffer. Protein was removed from the beads by boiling in 2X SDS loading buffer for 5 min and separated by SDS-PAGE, followed by western blotting, probed with phospho-ERK1/2 antibodies, or as indicated.

### Statistical analysis

Statistically significant differences were calculated using the Student's *t*-test (SigmaPlot 10.0, Jandel Scientific, and San Rafael, CA, USA). Significance was set at *p* < 0.05. The values are the means ± standard deviation (SD) of at least three independent experiments.
